# A Look at Spinal Cord Injury in Canada: Rick Hansen Spinal Cord Injury Registry (RHSCIR) – 2021 SCI Data Summary

**DOI:** 10.46292/sci23-00031S

**Published:** 2023-11-17

**Authors:** Vanessa K. Noonan

**Affiliations:** 1 Praxis Spinal Cord Institute, Vancouver, British Columbia, Canada

**Keywords:** cause of injury, incidence, prevalence, Rick Hansen Spinal Cord Injury Registry, secondary complications, severity and level of injury, spinal cord injury

## Abstract

The Rick Hansen Spinal Cord Injury Registry (RHSCIR) is a prospective registry of individuals who sustain a spinal cord injury (SCI) from 18 acute and 14 rehabilitation (rehab) Canadian hospitals specializing in SCI care. The data summary provides demographic and clinical details on 1148 people with either a traumatic spinal cord injury (tSCI) or a nontraumatic spinal cord injury (ntSCI) who were treated at a RHSCIR hospital in 2021. Information about the patient demographics, cause and severity of injury, care pathway, length of hospital stay, secondary complications, and social impacts after SCI were included. Data from the summary can provide researchers, healthcare providers, and decision makers with knowledge and evidence that may support strategies to improve SCI care services within their institutions.

This data summary provides brief demographic and clinical details on people who sustained a traumatic spinal cord injury (tSCI) or nontraumatic spinal cord injury (ntSCI) in 2021 in Canada. The Rick Hansen Spinal Cord Injury Registry (RHSCIR) is a prospective registry of individuals with a new SCI from 18 acute and 14 rehabilitation (rehab) hospitals specializing in SCI care in Canada. With 30 participating facilities from across Canada, it includes over 10,000 participants, making it the largest registry that tracks the experiences of individuals living with SCI in Canada. For previous reports and more details, visit https://praxisinstitute.org/research-care/key-initiatives/national-sci-registry/.

## Incidence

In 2021, there were 682 tSCI and 466 ntSCInew RHSCIR participants. As the collection of ntSCI was implemented across registry sites in a phased manner, the data collected in 2021 was not a full representation of the volume of ntSCI admitted to RHSCIR rehab sites. RHSCIR captures 60% to 70% of all acute traumatic SCI in Canada when compared to other national data sources (Canadian Institute for Health Information).^1^

## Prevalence

In Canada, of the 86,000 individuals living with SCI, it was estimated that approximately 30,000 people live with tSCI.^2^,^3^ Although SCI affects fewer individuals when compared to other chronic conditions, the economic burden is substantial. For people with tSCI, it was estimated that approximately 1100 people were discharged from hospital with a tSCI each year, and the estimated average lifetime cost is $2 million per individual.^3^,^4^ This includes direct costs like hospital stay and indirect costs such as lost productivity due to premature mortality.

## Demographics

The average age at injury was 55 years old for participants with tSCI and 60 years old for participants with ntSCI. Seventy-nine percent of participants with tSCI and 58% of participants with ntSCI were male.

## Cause of Injury

The most common type of traumatic injury was falls, followed by transportation, sports, others causes of injury (e.g., work-related injuries), and assault. The average age for people who experienced a fall was 63 years old; for those who experienced a transportation injury, it was 47 years old.

The most common nontraumatic cause was degenerative disease, followed by tumour, infection, and other nontraumatic cause, such as spinal hematomas, vascular disorders, inflammation, and congenital/genetic disorder. The average age for people who experienced a degenerative disease was 63 years old; for those who experienced a tumour, it was 59 years old.

## Severity and Level of Injury

**Figure 1.**
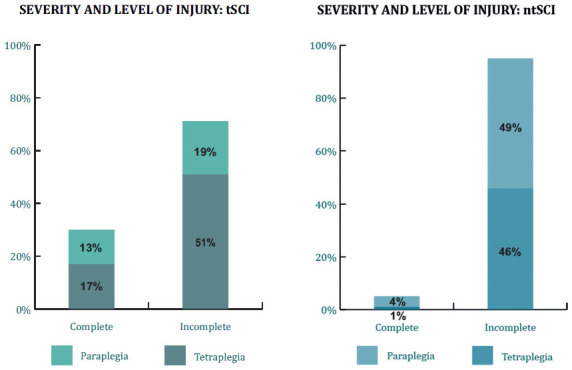
Severity and level of injury of participants with tSCI and ntSCI.

Tetraplegia was more common than paraplegia among participants with tSCI. Meanwhile, paraplegia was slightly more common among participants with ntSCI (**Figure 1**).

Incomplete injuries were more common than complete injuries in both participants with tSCI and ntSCI. For individuals over 65 years old, incomplete tetraplegia was more prevalent in tSCI and had a similar incidence to incomplete paraplegia for participants with ntSCI.

## Admission Locations

Seventy-seven percent of participants with tSCI were admitted to a SCI-specialized acute hospital within 24 hours from injury regardless of whether they first went to a nonspecialized hospital. Sixty-three percent of individuals with tSCI were admitted to a SCI-specialized acute hospital then went to a SCI-specialized rehab hospital before returning to the community. Mortality during the initial SCI-specialized acute hospital stay was 5% for participants with tSCI.

## Length of Stay

Average acute length of stay (LOS) was 44 days for individuals with tSCI with complete tetraplegia, 28 days for those with incomplete tetraplegia, 34 days for those with complete paraplegia, and 23 days for those with incomplete paraplegia.

For individuals with tSCI who were admitted to a SCI-specialized rehab hospital, the average LOS was 112 days for those with complete tetraplegia, 82 days for those with incomplete tetraplegia, 73 days for those with complete paraplegia, and 65 days for those with incomplete paraplegia.

For individuals with ntSCI who were admitted to a SCI-specialized rehab hospital, the average LOS was 95 days for those with complete tetraplegia, 67 days for those with incomplete tetraplegia, 70 days for those with complete paraplegia, and 55 days for those with incomplete paraplegia.

## Change in Employment Status, Household Income, and Relationship Status Five Years Post tSCI

Five-year post-tSCI data were collected for participants who completed follow-up questionnaires between 2018 and 2021. The mean age at injury of the participants completing the follow-up questionnaires was 47, and 78% were male respondents.

**Figure 2.**
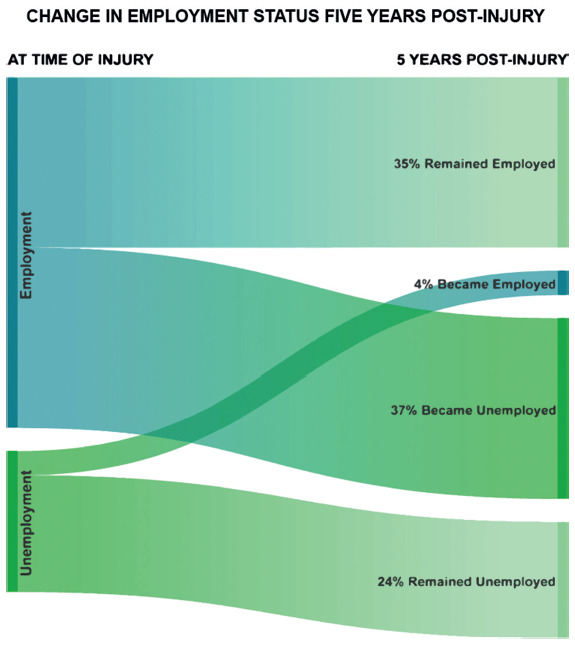
Change in employment status five years post tSCI.

Five years after injury, 35% of participants remained employed and 4% became employed, whereas 37% of participants became unemployed and 24% remained unemployed (**Figure 2**).

**Figure 3.**
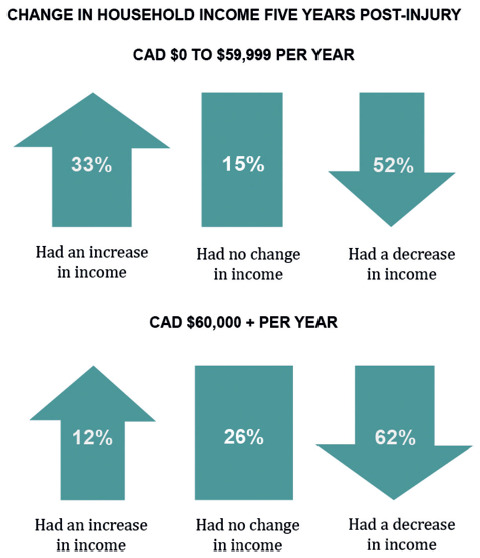
Change in household income five years post tSCI.

For participants making over CAD $60,000 per year, 12% had an increase in income, however 62% had a decrease in income five years after injury (Figure 3). The income was split at CAD $60,000 to match the median Canadian income in 2016 (https://www150.statcan.gc.ca/n1/daily-quotidien/220323/t002a-eng.htm). The median income has since increased but has been kept for annual comparisons.

**Figure 4.**
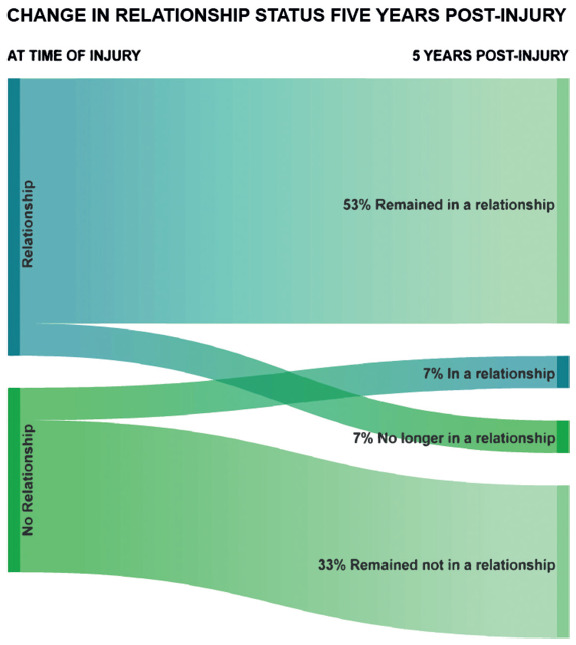
Change in relationship status five years post tSCI.

Relationship status did not appear to be significantly impacted five years after injury (**Figure 4**).

## Frequency of Secondary Complications

**Figure 5.**
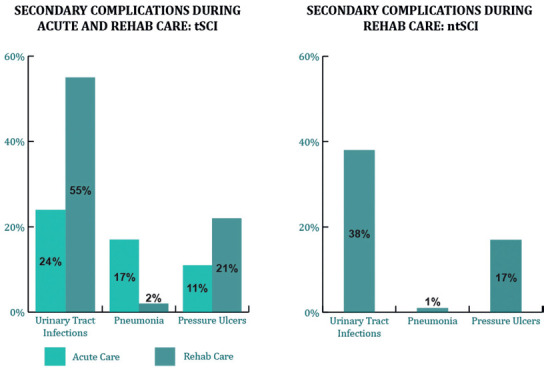
Secondary complications during acute and/or rehab care for participants with tSCI and ntSCI.

Urinary tract infections were the most common secondary complication in individuals with tSCI during acute and/or rehab admission and in individuals with ntSCI during rehab admission. For individuals with tSCI in acute care, pneumonia was the next common, followed by pressure injuries. For individuals with tSCI and ntSCI in rehab, pressure injuries were the next common, followed by pneumonia. Fifty-six percent of individuals with tSCI reported the occurrence of at least one of the secondary complications, and 20% reported multiple secondary complications during acute and/or rehab admission. Besides, 47% of individuals with ntSCI reported having at least one of the secondary complications, and 8% had multiple secondary complications during admission (**Figure 5**).

## References

[1] Noonan VK, Chan E, Santos A, RHSCIR Network (2017). Traumatic spinal cord injury care in Canada: A survey of Canadian centers. J Neurotrauma.

[2] Noonan VK, Fingas M, Farry A, Baxter D, Singh A, Fehlings MG, Dvorak MF (2012). Incidence and prevalence of spinal cord injury in Canada: A national perspective. Neuroepidemiology.

[3] Thorogood NP, Noonan VK, Chen X, Fallah N, Humphreys S, Dea N, Kwon BK, Dvorak MF (2023). Incidence and prevalence of traumatic spinal cord injury in Canada using health administrative data. Front Neurol.

[4] Krueger H, Noonan VK, Trenaman LM, Joshi P, Rivers CS (2013). The economic burden of traumatic spinal cord injury in Canada. Chronic Dis Inj Can.

